# SPIO Enhance the Cross-Presentation and Migration of DCs and Anionic SPIO Influence the Nanoadjuvant Effects Related to Interleukin-1β

**DOI:** 10.1186/s11671-018-2802-0

**Published:** 2018-12-20

**Authors:** Hui Liu, Heng Dong, Na Zhou, Shiling Dong, Lin Chen, Yanxiang Zhu, Hong-ming Hu, Yongbin Mou

**Affiliations:** 1 Central Laboratory, Nanjing Stomatological Hospital, Medical School of Nanjing University, #30 Zhongyang Road, Nanjing, 210008 Jiangsu China; 2 Laboratory of Cancer Immunobiology, Robert W. Franz Cancer Research Center, Earle A. Chiles Research Institute, Providence Cancer Center, Portland, OR USA

**Keywords:** Superparamagnetic iron oxide nanoparticles, Dendritic cells, Cross-presentation, Surface charge, Interleukin-1β, Nanoadjuvant

## Abstract

Superparamagnetic iron oxide nanoparticles (SPIO) have been synthesized and explored for use as carriers of various nanoadjuvants via loading into dendritic cells (DCs). In our study, homogeneous and superparamagnetic nanoparticles are susceptible to internalization by DCs and SPIO-pulsed DCs showed excellent biocompatibility and capacity for ovalbumin (OVA) cross-presentation. Herein, we found that SPIO-loaded DCs can promote the maturation and migration of DCs in vitro*.* SPIO coated with 3-aminopropyltrimethoxysilane (APTS) and meso-2,3-dimercaptosuccinic acid (DMSA), which present positive and negative charges, respectively, were prepared. We aimed to investigate whether the surface charge of SPIO can affect the antigen cross-presentation of the DCs. Additionally, the formation of interleukin-1β (IL-1β) was examined after treatment with oppositely charged SPIO to identify the nanoadjuvants mechanism. In conclusion, our results suggest that SPIO are biocompatible and can induce the migration of DCs into secondary lymph nodes. SPIO coated with APTS (SPIO/A^+^) exhibited excellent adjuvant potentials for the promotion of antigen cross-presentation and T cell activation and surpassed that of DMSA-coated nanoparticles (SPIO/D^−^). This process may be related to the secretion of IL-1β. Our study provides insights into the predictive modification of nanoadjuvants, which will be valuable in DC vaccine design and could lead to the creation of new adjuvants for applications in vaccines for humans.

## Background

Adjuvants have been applied extensively for clinical and experimental use, and they have long been considered as either immune-stimulatory agents, passive depots, or vehicles that are capable of promoting the required immune responses [1, 2]. In recent decades, many diverse classes of compounds have been used as adjuvants, including nanoparticles, microbial products, emulsions, cytokines, polymers, and liposomes [3–5]. The evolution of nanoparticles as immune adjuvants (nanoadjuvants) represents a remarkable area of antigen delivery based on magnifying the immune responses. Among all types of nanoparticles, superparamagnetic iron oxide nanoparticles (SPIO) have great biocompatibility, proper surface architecture, and flexible ligand conjugation [6]. These properties make them applicable in many different biomedical areas, such as magnetic resonance imaging (MRI), targeted drug delivery, and hyperthermia therapy [7, 8].

Dendritic cells (DCs), the major professional antigen-presenting cells (APCs), play a critical role in cell-mediated adaptive immunity in which immunological memory is generated after a primary response to a specific antigen, and this memory leads to an enhanced response to subsequent encounters with that antigen [9, 10]. In addition, DCs can promote the presentation of exogenous antigens by major histocompatibility complex class I (MHC-I), a process called cross-presentation, and then activate cytotoxic T lymphocytes (CTLs) [11]. Soluble antigens destined for cross-presentation are internalized by receptor-mediated endocytosis and then transferred to the cytoplasm for proteasomal degradation and peptide loading. Conventional DC vaccines lead to a moderate immune response due to the relatively unsatisfactory transport of soluble antigens. Therefore, nanoadjuvants have been explored as carriers of soluble antigens to enhance cross-presentation in DCs in many research studies [12–14].

Coating materials play critical roles in the stabilization and subsequent functionalization of aqueous SPIO suspensions [15]. In previous studies, positively charged SPIO were shown to facilitate the cross-presentation ability of DCs to enhance the immune response, and they surpassed the effect of their oppositely charged counterparts [16, 17]. The mechanism through which differently charged SPIO can affect the antigen cross-presentation of DCs has not been clarified. Interleukin-1β (IL-1β), a prototypic proinflammatory cytokine that participates in innate immunity, can be secreted by immune cells, such as DCs, when sensing pathogen-associated molecular patterns (PAMPs) and damage-associated molecular patterns (DAMPs) [18]. The production of IL-1β is strictly mediated by inflammasomes, especially NLRP3 (NACHT, LRR, and PYD domains-containing protein 3). Activation of NLRP3 induces the production of caspase-1, which then cleaves inactive pro-IL-1β to the active form IL-1β [19]. Certain inorganic nanoparticles, such as silica, double-walled carbon nanotubes, and titanium dioxide, can induce inflammasome formation [20–22]. Previous studies have reported a correlation between the surface charge of magnetite nanoparticles and their cellular efficiency [23]. Herein, we speculate that different surface charges on SPIO-loaded DCs may also influence the secretion of IL-1β, and our objective is to investigate the relationship between these surface charges and the cross-presentation function of DCs.

## Methods and Materials

### Preparation of the SPIO

To prepare SPIO, an easy coprecipitation method was employed as previously reported [24]. In brief, a mixed solution of FeCl_3_ and FeSO_4_ (molar ratio Fe^3+^:Fe^2+^ = 2:1) was prepared under nitrogen atmosphere and energetically stirred at 37 °C for 30 min. A black-colored precipitate of Fe_3_O_4_ nanoparticles was formed and immediately washed five times with distilled water using magnetic separation. The Fe_3_O_4_ was then dispersed in distilled water to a concentration of 3 mg/mL at a pH of 3. Ultimately, the suspension was aerated (with air) at 95 °C and the brown SPIO were separated out.

SPIO coated with DMSA (SPIO/D^−^) and SPIO coated with APTS (SPIO/A^+^) were prepared by coating SPIO with meso-2,3-dimercaptosuccinic acid (DMSA) and 3-aminopropyltrimethoxysilane (APTS), respectively. For SPIO/D^−^, an aqueous solution of DMSA at a molar ratio of 1:40 was added to 100 mL of SPIO solution. After a 4-h reaction with continuous stirring, the SPIO/D^−^ was separated out at a rate of 500 rpm at 50 °C. For SPIO/A^+^, APTS was added to the SPIO solution at a molar ratio of 0.2:1 with vigorous stirring for 5 h. Then, the precipitate was dissolved out with a permanent magnet and washed with deionized water, and the solution was treated using ultrasonic waves. The resulting solution was washed repeatedly with water, and the SPIO/A^+^ were finally dried into a powder at 37 °C under vacuum.

### Mice

C57BL/6 mice and enhanced green fluorescent protein (EGFP)-transgenic C57BL/6 mice were purchased from the Model Animal Research Center of Nanjing University and housed in specific pathogen-free (SPF) conditions at the Central Laboratory, Nanjing University. All animal experiments were performed in accordance with protocols approved by the Animal Care and Use Committee of the Medical School, Nanjing University, China.

### Cell Culture

Murine DCs were generated from mouse bone marrow as previously described [25]. In brief, bone marrow monocytes of 8-week-old C57BL/6 mice were separated from their femurs and tibias. Subsequently, the cells were cultured in RPMI-1640 (Gibco, Thermo Fisher Scientific, Waltham, MA, USA) along with 10% fetal bovine serum (FBS), 10 ng/mL murine granulocyte-macrophage colony-stimulating factor (GM-CSF; Gibco, USA), and 1 ng/mL murine interleukin-4 (IL-4, PeproTech, Rocky Hill, NJ, USA). The culture medium was replaced with fresh medium every 2 days. Immature DCs were generally collected on day 6. The EGFP-DCs were derived from EGFP-transgenic C57BL/6 mice according to the method mentioned above.

Thawed frozen human peripheral blood mononuclear cells (PBMCs) were rested overnight in complete medium, i.e., X-VIVOTM 15 (Lonza Group Ltd., Basel, Switzerland) = 1:1. After density gradient centrifugation, the PBMCs were suspended in medium for 4 h. Human DCs from the adherent cells were cultured at 37 °C in medium containing human GM-CSF (100 ng/mL; R&D Systems, Minneapolis, MN, USA) and human IL-4 (10 ng/mL; PeproTech, Rocky Hill, NJ, USA). Half of the medium was changed on days 2 and 4. Immature human DCs were harvested on day 5. Cytomegalovirus Epstein-Barr virus and influenza virus (CEF)-specific T cells were derived from suspended cells, which were cultured in CMX media containing MHC-I restricted CEF peptide (20 ng/mL, Panatecs, Heilbronn, Germany, PA-CEF-002) for approximately 48 h. CEF-specific T cells were expanded by 1000  U/mL human IL-2 (Peprotech, Rocky Hill, NJ, USA, 200–02) in CMX medium for 12 days. For IL-2, the medium was replaced with fresh medium every 3 days.

### Characterization

The morphology and size of the SPIO were determined using a transmission electron microscope (TEM, Advanced Microscopy Techniques, Danvers, MA). An X-ray diffraction (XRD) pattern was used to identify the spectra of the catalyst. The zeta potential assay was also measured to determine the surface charges of the nanoparticles coated with different polymers. The SPIO/A^+^ and SPIO/D^−^ nanoparticles were prepared with pH values ranging from 3 to 8. Zeta potential measurements were made with a zetasizer Nano ZS90 potential analyzer (Malvern, UK). The magnetic properties of the nanoparticles were analyzed at 37 °C using a vibrating sample magnetometer (Lakeshore 7407).

### CPRG Assay

The B_3_Z T-cell line (a CD8^+^ T cell hybridoma) can express the LacZ gene when its T cell receptor engages an ovalbumin (OVA) 258–265 epitope in the presence of the H-2Kb MHC-I molecule. DCs (2 × 10^4^) and OVA (100 μg/mL, Sigma-Aldrich) were cultured with SPIO, SPIO/A^+^, or SPIO/D^−^ at 37 °C. Six hours later, the DCs were cocultured with B_3_Z (2 × 10^5^) overnight. A chlorophenol red-β-galactosidase assay (CPRG, Sigma-Aldrich, USA) was conducted to determine the β-galactosidase production of the B_3_Z cells. In this assay, the optical density (OD) at 595 nm indicates the antigen cross-presentation ability of the DCs.

### Cells Apoptosis Assay

Fluorescence-activated cell sorting (FCS) was employed to explore the effects of different concentrations of SPIO on the viability of DCs. In brief, immature DCs were incubated with SPIO marked with Annexin V and PI (Biouniquer, CHN), and the expression of Annexin V and PI in the DCs were examined via FCS.

### Fluorescence Intensity Imaging In Vivo

To explore the migration of DCs in vivo, TNF-α (60 ng/mouse) was preinjected into the footpads of both hind legs (*n* = 8). After 24 h, SPIO-labeled EGFP-DCs (2 × 10^6^) in 40 μL of phosphate-buffered saline (PBS) were injected into the left footpads of C57BL/6 mice, and the same number of unlabeled EGFP-DCs was injected into the right side. To examine the level of EGFP-DCs migrating to the lymph nodes, a Maestro imaging system (CRi, Woburn, MA, USA) was employed. The dissected lymph nodes were observed using 484 nm excitation filters and 507 nm emission filters. The fluorescent images displaying green fluorescence were then analyzed with Living Image software (v 2.50; Caliper Corporation, Newton, MA, USA). Confocal microscopy analyzes were used to determine the immunohistochemistry. Frozen lymph nodes were cut into 5-μm-thick sections and then fixed, and the sections were incubated with green fluorescent protein (GFP) antibody (Invitrogen), with goat Alexa Fluor 488 nm antibody (Invitrogen) used as the secondary antibody. A confocal laser scanning microscope (Fluoview, Fv10i; Olympus) was used to observe the samples.

### Human DC Antigen Cross-Presentation Assay In Vitro

The cultured human DCs (2 × 10^4^) were seeded into a 96-well round-bottomed plate to which SPIO/A^+^ and SPIO/D^−^ nanoparticles (100 μg/mL) combined with cytomegalovirus (CMV) pp65 protein (1 μg/mL, MACS) were added, and the CMV pp65 protein and CEF peptide (1 μg/mL, Think peptides) were used separately as controls. After 6 h, CEF-specific T cells (2 × 10^5^) were added to the human DCs for 12 h. Brefeldin A (10 μg/ml, Sigma-Aldrich, USA) was kept in the medium for another 6 h. After stimulation and activation, CEF-specific T cells were gathered and stained with human antibodies against CD3, CD4, CD8, and IFN-γ (Invitrogen, USA). After staining, the samples were analyzed by a custom-built LSR II flow cytometer (BD, Franklin Lakes, NJ, USA). The collected data were analyzed by FlowJo software (Tree Star, Ashland, OR, USA).

### Enzyme-Linked Immunosorbent Assay

A mouse IL-1β enzyme-linked immunosorbent assay (ELISA) Ready SET-Go kit (eBioscience, USA) and a human IL-1β ELISA kit (Thermo Fisher Scientific, Waltham, MA, USA) were employed to determine the secretion of IL-1β by DCs. An ELISA plate (Costar, USA) coated with 100 μL/well of IFN-γ was used to capture antibodies overnight at 4 °C and blocked with ELISA buffer. Samples and standards were then added to the wells and incubated for 2 h at 37 °C. Biotinylated IFN-γ was detected. The samples were measured using a microplate reader with an OD setting of 450 nm (BioTek, USA).

### Statistical Analysis

The data were analyzed using the Statistical Package for Social Science (SPSS 13.0, Chicago, IL, USA). The results were presented as the means ± SD, and differences between the control and test groups were assessed by a one-way analysis of variance, two-tailed Student’s *t* tests, and double-factor analysis of variance. Differences at *P < 0.05 were considered statistically significant.

## Results

### Characterization of SPIO

**Fig. 1:**
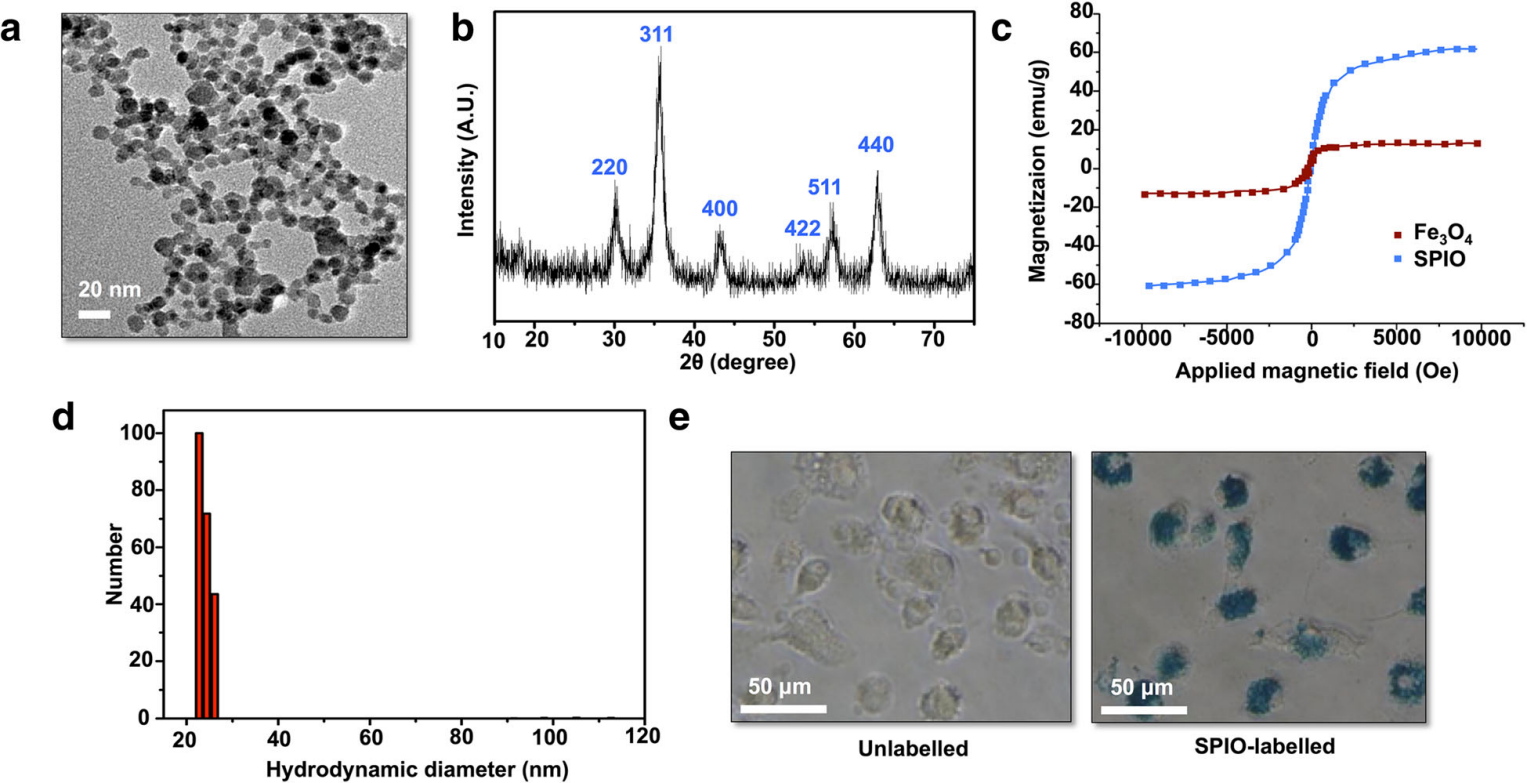
Characterization of SPIO. **a** TEM image of the obtained SPIO. **b** XRD pattern of the nanoparticle catalyst indicating that the material is γ-Fe_2_O_3_. **c** Magnetization curves of the obtained SPIO and Fe_3_O_4_ nanoparticles. **d** Hydrodynamic diameter of SPIO. **e** Morphology of the DCs labeled with 50 μg/mL SPIO after 12 h of incubation: unlabeled DCs and Prussian blue stain-labeled DCs

The morphology and size of the synthesized SPIO were observed via TEM. The TEM image showed that SPIO had a mean size of 8.7 nm and a spherical shape (Fig. 1a). The XRD analysis showed six peaks that distinctly matched the standard γ-Fe_2_O_3_ reflections (Fig. 1b). The vibrating magnetometer results demonstrated that the obtained SPIO possessed superparamagnetic behavior, with a saturation magnetization of 60.4 emu/g better than Fe_3_O_4_ (Fig. 1c). The DLS plot showed that the size distribution of the SPIO is 22 nm in solution (Fig. 1d). To confirm that the DCs contained SPIO, Prussian blue staining was performed to verify that the DCs contained iron (Fig. 1e).

### SPIO Activated Cross-Presentation of DCs

**Fig. 2:**
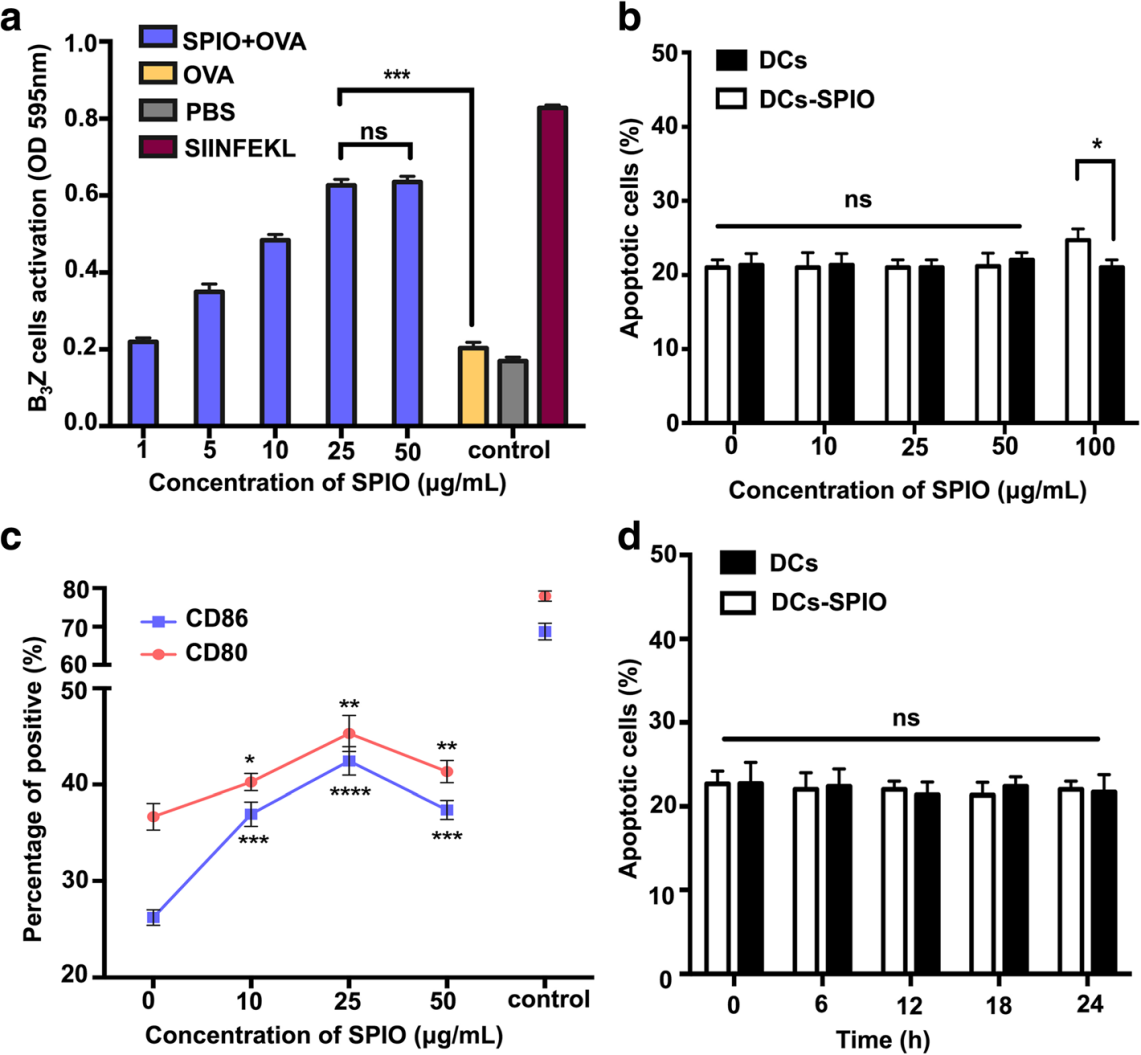
Influences of DC cross-presentation and biocompatibility after labelling with SPIO. **a** Cross-presentation of DCs enhanced at different SPIO concentrations. **b** Apoptotic DCs and SPIO-labeled DCs examined by FCS at different concentrations (10, 25, 50, and 100 μg/mL). **c** Phenotypes of the DCs, including CD11c^+^CD80^+^ and CD11c^+^CD86^+^ after labelling with SPIO (10, 25, 50 μg/mL). DCs stimulated with LPS (1 μg/mL) were used as positive controls. **d** Apoptotic DCs loaded with 25 μg/mL SPIO examined by FCS at different time points (6, 12, 18, and 24 h)

To further study the effect of SPIO-labeled DCs on T cell activation in a murine system, the level of B_3_Z T-cell activation was determined by examining the production of β-galactosidase by CPRG assay. A fixed concentration of 100 μg/mL OVA and five dose ratios of SPIO (1, 5, 10, 25, and 50 μg/mL after 6 h) were adopted in this study. As the concentration of SPIO increased, the degree of activation of B_3_Z cells increased gradually and reached stability at 25 μg/mL (Fig. 2a). To investigate whether the viability of DCs labeled with the various concentrations of SPIO was influenced, the DCs and SPIO-labeled DCs were analyzed via FCS after Annexin V and PI staining. The results indicated that the total percentage of Annexin V/PI DCs at SPIO concentrations of 10, 25, and 50 μg/mL did not significantly differ, while the percentage of apoptotic cells increased after loading with 100 μg/mL SPIO (Fig. 2b). The surface costimulatory molecules of the DCs labeled with various concentrations of SPIO were observed by FCS. The expression of CD80 and CD86 had a detectable increase at 25 μg/mL compared with those without SPIO labeling (Fig. 2c). DCs labeled with 25 μg/mL SPIO did not exhibit changes in cell apoptosis at different time points (Fig. 2d). We used 25 μg/mL in the following experiments.

### Labelling EGFP-DCs with SPIO Enhanced EGFP Signals in Secondary Lymph Nodes

**Fig. 3:**
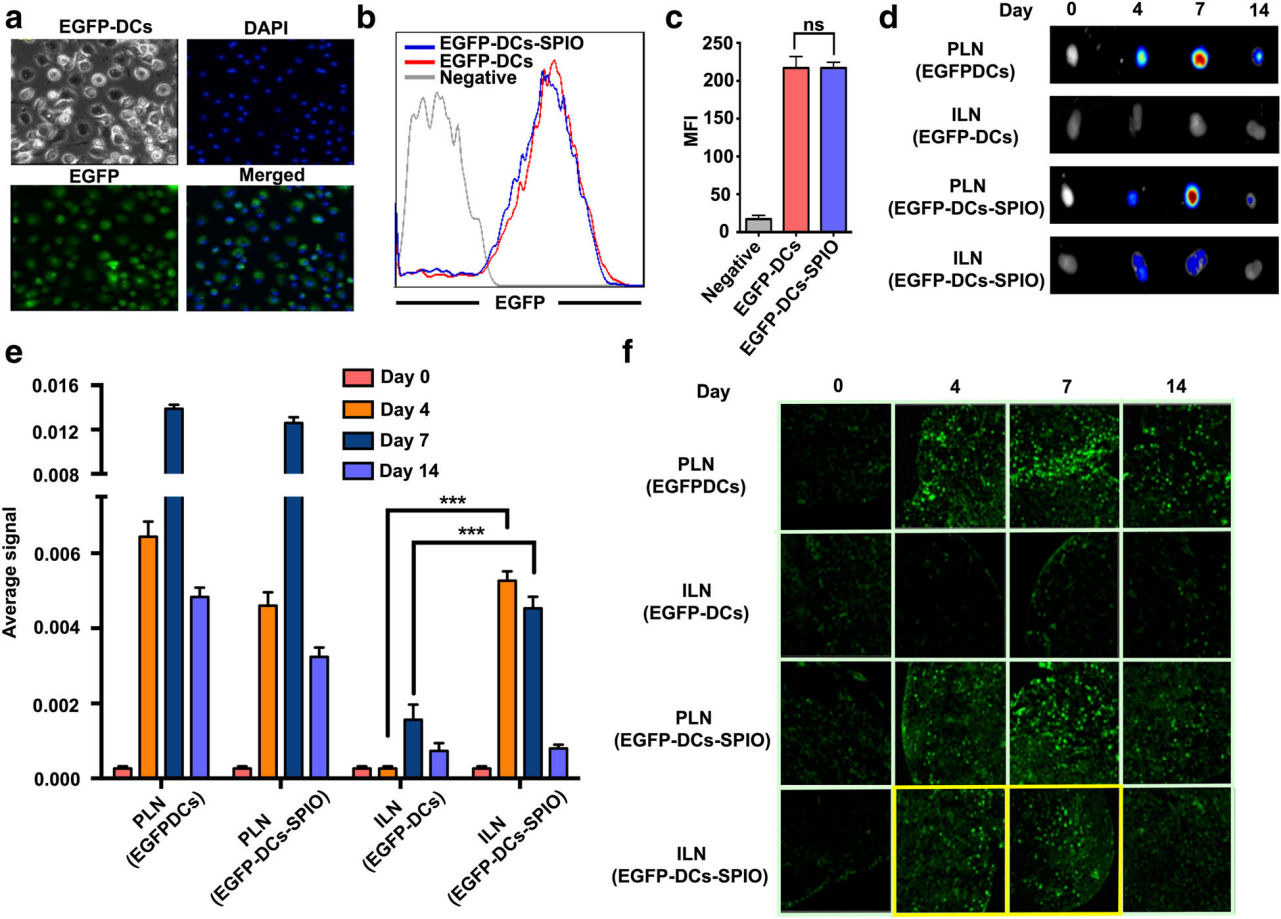
Migration and location of EGFP-DCs after labelling with SPIO. **a** Laser scanning confocal microscopy of EGFP-DCs labeled with 25 μg/mL SPIO nanoparticles after 12 h of incubation. **b**, **c** Fluorescence intensity of the SPIO-labeled EGFP-DCs after 12 h. In vitro optical imaging of drainage lymph nodes after the back transfusion of SPIO-labeled EGFP-DCs at different days. TNF-α was first injected into the mouse foot pad in advance to promote the migration of EGFP-DCs. **d**–**e** In vitro optical imaging and signal intensity analysis of lymph nodes on different days after injection of EGFP-DCs with or without SPIO. **f** EGFP positive cells of draining lymph nodes were detected by laser confocal microscopy

EGFP-DCs were successfully derived from EGFP-transgenic mice in vitro, and confocal fluorescence microscopy images showed that almost all EGFP-DCs exhibited green fluorescence (Fig. 3a). To investigate whether the green fluorescence of the EGFP-DCs could be affected by SPIO, FCS was conducted. The results showed that the expression of EGFP fluorescence was not weakened after SPIO labeling (Fig. 3b, c). Then, the 25 μg/mL SPIO-labeled EGFP-DCs were injected into the right-side hind footpads of C57BL/6 mice and unlabeled EGFP-DCs were injected into the opposite side. The EGFP signals were measured in the popliteal lymph node (PLN) and inguinal lymph node (ILN), which are the sentinel lymph nodes and secondary lymph nodes, respectively. The results showed that the migration of SPIO-labeled EGFP-DCs and unlabeled EGFP-DCs in the sentinel lymph nodes reached a peak on day 7. Significant differences in the EGFP signal were not observed between the two groups, whereas a significant reduction was detected in the PLN group on day 14. The EGFP signals detected in the ILN group were consistent with that of the SPIO-labeled EGFP-DC group on the 4th day and 7th day, which indicated that the SPIO-labeled DCs migrated to the secondary lymph nodes (Fig. 3d–f).

### Differently Charged Modified SPIO Affected Murine DCs Cross-Presentation

**Fig. 4:**
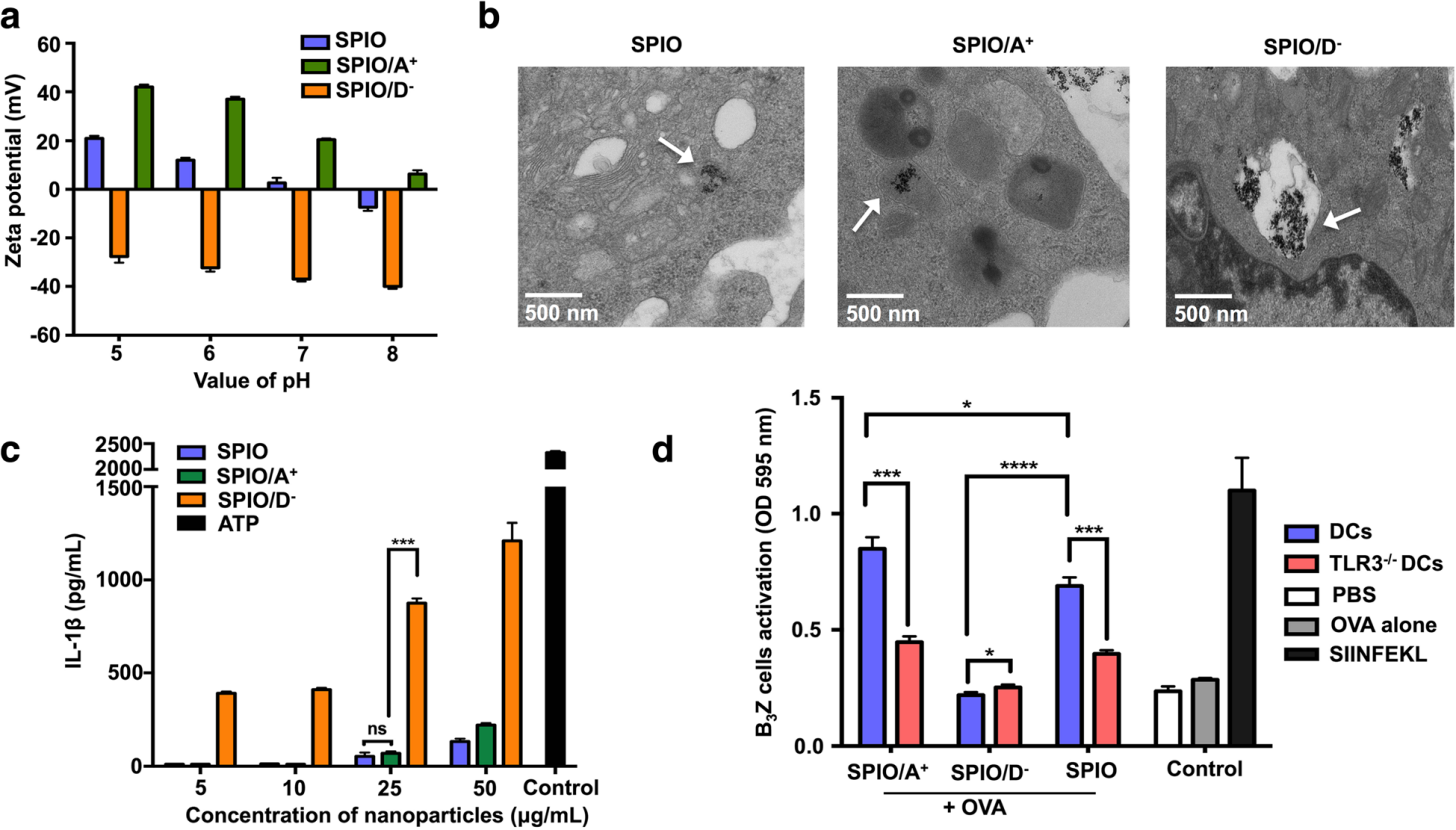
SPIO coated with differently charges affected DC cross-presentation and IL-1β secretion. **a** The pH-dependent zeta potential of SPIO coated with differently charged molecules. **b** Locations of nanoparticles with different charges in DCs under TEM. **c** IL-1β induced by SPIO coated with differently charged molecules. **d** Cross-presentation of SPIO/A^+^+OVA and SPIO/D^−^ + OVA by DCs through the TLR3 pathway

The SPIO were coated with APTS or DMSA. To verify the surface charge of the SPIO, we measured the zeta potential characteristic. The zeta potentials of the SPIO/A^+^ and SPIO were positive, whereas the zeta potential of the SPIO/D^−^ showed a negative charge in solution when the pH value was 7 (Fig. 4a). The ultrastructure of the SPIO-labeled DCs coated with differently charged polymers was observed via TEM. The DCs treated with SPIO appeared electron-dense compared with untreated cells, and numerous SPIO were clustered together in the cytoplasm. The SPIO/A^+^ were engulfed by endosomes in the cytoplasm, whereas a higher amount of SPIO/D^−^ were found in the cytoplasm of the DCs, and nearly all these nanoparticles were surrounded by several-layered membrane structures that resembled lysosomes (Fig. 4b). We examined whether the oppositely charged SPIO could trigger different levels of IL-1β. According to our results, DCs loaded with SPIO/A^+^ and SPIO/D^−^ induced a dose-dependent secretion of IL-1β. Regardless of the concentration, we found that SPIO/D^−^ induced significantly higher levels of IL-1β than SPIO/A^+^ (Fig. 4c). Due to the obvious correlation between IL-1β and the TLR3 pathway, to further study the effect of IL-1β on T cell activation, DCs were derived from TLR3 knockout mice and cocultured with SPIO/A^+^, SPIO/D^−^, and OVA. The DCs loaded with SPIO/A^+^ + OVA could effectively activate B_3_Z T cells, which means that the TLR3 molecule in the DCs was definitely necessary for cross-presentation (Fig. 4d).

### SPIO Modified with Oppositely Charged Coatings Promoted Antigen Cross-Presentation by Human DCs and Were Affected by IL-1β

**Fig. 5:**
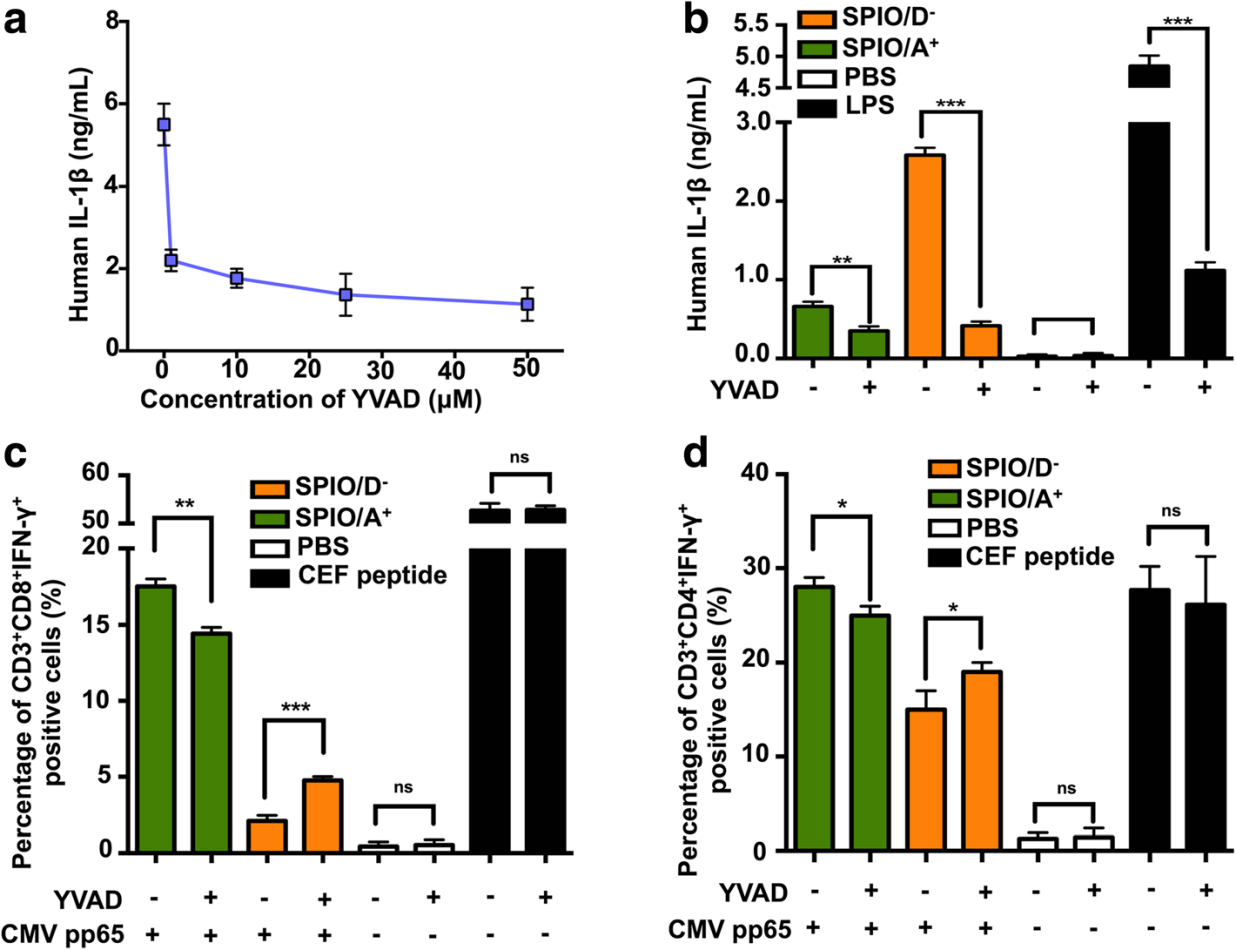
SPIO coated with opposite charges affect the function of human DCs via the IL-1β pathway. **a** After pre-treatment by LPS for 3 h, human DCs were incubated with increasing concentrations of YVAD (1, 10, 25, and 50 μM), and then the supernatants were collected for an ELISA of IL-1β. **b** YVAD can inhibit the secretion of IL-1β from human DCs via SPIO/D^−^. SPIO/A^+^, and SPIO/D^−^ affect the DC cross-presentation influenced by IL-1β. **c** CD3^+^CD8^+^IFN-γ^+^ and **d** CD3^+^CD4^+^IFN-γ^+^ T cells were analyzed by intracellular staining using FCS

To explore the relationship between IL-1β and cross-presentation, we selected the caspase-1 inhibitor YVAD and found that it could significantly inhibit the IL-1β secretion of human DCs after pretreatment with 50 μM lipopolysaccharide (LPS) for 3 h (Fig. 5a). In addition, we found that YVAD could inhibit the secretion of IL-1β from human DCs caused by SPIO/A^+^ and SPIO/D^−^ (Fig. 5b). We then investigated whether human DCs could be used as effective APCs and cross-present CMV pp65 to CEF-specific T cells. To measure the antigen cross-presentation, intracellular staining (ICS) was introduced to determine the percentage of antigen-specific CD3^+^CD8^+^IFN-γ^+^ and CD3^+^CD4^+^IFN-γ^+^ T cells by FCS. DCs loaded with SPIO/A^+^ combined with the CMV pp65 protein induced more CD3^+^CD8^+^IFN-γ^+^ and CD3^+^CD4^+^ IFN-γ^+^ T cells than DCs loaded with SPIO/D^−^. Our data showed that the caspase-1 inhibitor YVAD significantly increased the T cell responses induced by the SPIO/D^−^ + CMV pp65 protein, whereas it partly inhibited the T cell responses induced by the SPIO/A^+^ + CMV pp65 protein (Fig. 5c, d). These results indicated that a moderate level of IL-1β activation is needed for efficient cross-presentation and that a high level of IL-1β activation suppresses cross-presentation in DCs.

## Discussion

Immunotherapy has been a research focus in clinical and experimental studies since the development of biotechnology. However, previous clinical trials of traditional DC vaccines designed to elicit immunity have not induced sufficient immune responses [26]. Nanoparticles represent a type of adjuvant, and can promote the function of DCs to activate T cells [27]. The most characteristic features of our SPIO include their biocompatibility and their application of labeling DCs as an immune adjuvant. The TEM images show that our SPIO have a mean size of 8.7 nm in the dry state and a spherical shape (Fig. 1a). Prussian blue staining demonstrated that the SPIO are susceptible to be phagocytized by DCs (Fig. 1e), thus indicating the ingestion efficiency.

SPIO have been reported to have broad biomedical applications, such as in cellular labeling, drug delivery, MRI, and magnetic hyperthermia [28], which are all applications that require biocompatibility. Therefore, whether DCs labeled with SPIO have an influence on DC apoptosis must be investigated. Our data suggested that there was no significant difference in DC apoptosis when DCs were labeled with less than 50 μg/mL SPIO (Fig. 2b). In the following study, we chose OVA protein as a model protein and B_3_Z T cells as effective T cells to observe whether SPIO can affect the cross-presentation of DCs. Our results indicate that SPIO-labeled DCs can markedly facilitate the cross-presentation of OVA and the activation of B_3_Z T cells. The surface molecules CD80 and CD86, which are markers of mature DCs, are two critical costimulatory factors necessary for T cell activation. At 25 μg/mL, the activated B_3_Z T cells increased with the nanoparticle dose and eventually stabilized (Fig. 2a). Moreover, the expression of CD80 and CD86 on the surface of DCs reached a maximum (Fig. 2c). Therefore, we adopted the concentration of 25 μg/mL and found that the apoptosis of DCs is time independent (Fig. 2d), which proved the excellent nanoadjuvant functions and biocompatibility of SPIO.

To examine the influence of SPIO on DC migration, we used EGFP-DCs, which presented green fluorescence under the confocal fluorescence microscopy, to coculture with 25 μg/mL SPIO for 12 h. We observed that the expression of EGFP fluorescence did not weaken after SPIO labeling. Ultimately, the migration of DCs to the secondary lymphoid organs is the key parameter for assessing the effectiveness of DC-based vaccines. In our previous study, over the first 24 h, SPIO-labeled DCs were not detected in a significant quantity in the secondary lymph nodes [29]. To further determine whether this phenomenon changed with time, both labeled and unlabeled EGFP-DCs were collected and injected into the foot pads of mice to evaluate the potential of SPIO in the migration of DCs. Our results showed that green fluorescence appeared in the ILN on days 4 and 7 (Fig. 3d–f) thus demonstrating that SPIO can facilitate the migration of EGFP-DCs to the secondary lymph nodes. This property can be applied to restrict the metastasis of tumors and activate a greater amount of CTL cells in more lymph nodes.

In our study, we have explored the adjuvant properties of oppositely charged SPIO and the possible mechanism underlying the changes in the function of DCs. The surface charge of the iron oxide nanoparticles has been shown to have influence on the cellular uptake efficiency [30, 31]. In our previous research, we reported that cationic SPIO could enhance antigen cross-presentation and, accordingly, T cell activation, while anionic SPIO were associated with autophagy. [16] Several metal nanoparticles can induce inflammatory responses [32, 33]. Iron oxide nanoparticles have been reported to activate NLRP3, rupture the membranes of lysosomes, release cathepsin B, and induce IL-1β secretion [34]. Thus, we hypothesized that the oppositely charged SPIO nanoparticles could serve different functions in stimulating the production of IL-1β in both murine and human DCs. In Fig. 4a, our zeta potential analysis showed that APTS-coated SPIO carried positive charges, whereas DMSA-coated SPIO carried negative charges. Under the TEM, we found that differently charged SPIO were clustered in different positions in the cytoplasm (Fig. 4b). To investigate the difference between the cross-presentation of SPIO/A^+^ and SPIO/D^−^, we explored IL-1β secretion in murine DCs induced by differently charged nanoparticles. We assessed the responses of murine DCs after treatment with SPIO/A^+^ and SPIO/D^−^. Exposure to SPIO/D^−^ induced the overt activation of IL-1β secretion compared with exposure to SPIO/A^+^ (Fig. 4c). This phenomenon partly revealed that the antigen cross-presentation induced by SPIO/D^−^ is not as efficient as that induced by SPIO/A^+^. In addition to the CPRG analysis of increasing B_3_Z T cell activation after the coculture of murine DCs with SPIO/A^+^+OVA demonstrated that positively charged nanoparticles can perform better when used as immune adjuvants. Toll-like receptors (TLRs) are of importance for triggering immune responses, such as TLR3, and they can ultimately result in the production of inflammasomes, the activation of caspase-1 protein, and secretion of cytokine IL-1β [35]. To demonstrate whether TLR3 is related to the antigen cross-presentation influenced by our nanoadjuvant, TLR3 knockout DCs were employed in our study. TLR3^−/−^ DCs loaded with SPIO/A^+^+OVA and SPIO + OVA effectively stimulated the B_3_Z T cell activation (Fig. 4d), which indicates that the TLR3 molecule in DCs was necessary for cross-presentation. Collectively, these results elucidated that differently charged polymers on the surface of nanoparticles should be considered when investigating their synergistic effects on activated immune responses.

Our data show that the oppositely charged nanoparticles could induce the production of IL-1β, whereas SPIO/D^−^ could hyperactivate the secretion of IL-1β. However, compared with SPIO/A^+^, SPIO/D^−^ induced a lower level of B_3_Z T cell activation. Therefore, we speculate that aberrant IL-1β production may contribute to cellular dysfunction in DCs. Most studies on nanomaterial adjuvants exploit DCs from mice, whereas few have used DCs from humans [36, 37]. To further verify our hypothesis, we used YVAD, which has been proven to be an effective caspase-1 inhibitor (Fig. 5a, b), to suppress IL-1β secretion from human DCs. CMV pp65 protein was selected as the model antigen, and CEF-specific T cells expanded in vitro were used as responder cells. The CMV pp65 protein is an immunological dominant protein that readily activates CD8^+^ and CD4^+^ T cells to produce cytokines, particularly IFN-γ [38]. CEF peptide was used as a positive control to verify that the addition of YVAD would not affect the activation of T cells because it can be presented to the T cells directly. Intriguingly, with the use of YVAD, the T cell responses in the SPIO/A^+^ group were slightly restrained, while the responses in the SPIO/D^−^ group increased (Fig. 5c, d). The influence of IL-1β on DC cross-presentation provided conclusive evidence that a low level of IL-1β induced by SPIO/A^+^ is needed for antigen cross-presentation, and a high level of IL-1β in the cytosol will inhibit the function of DCs.

## Conclusions

**Fig. 6:**
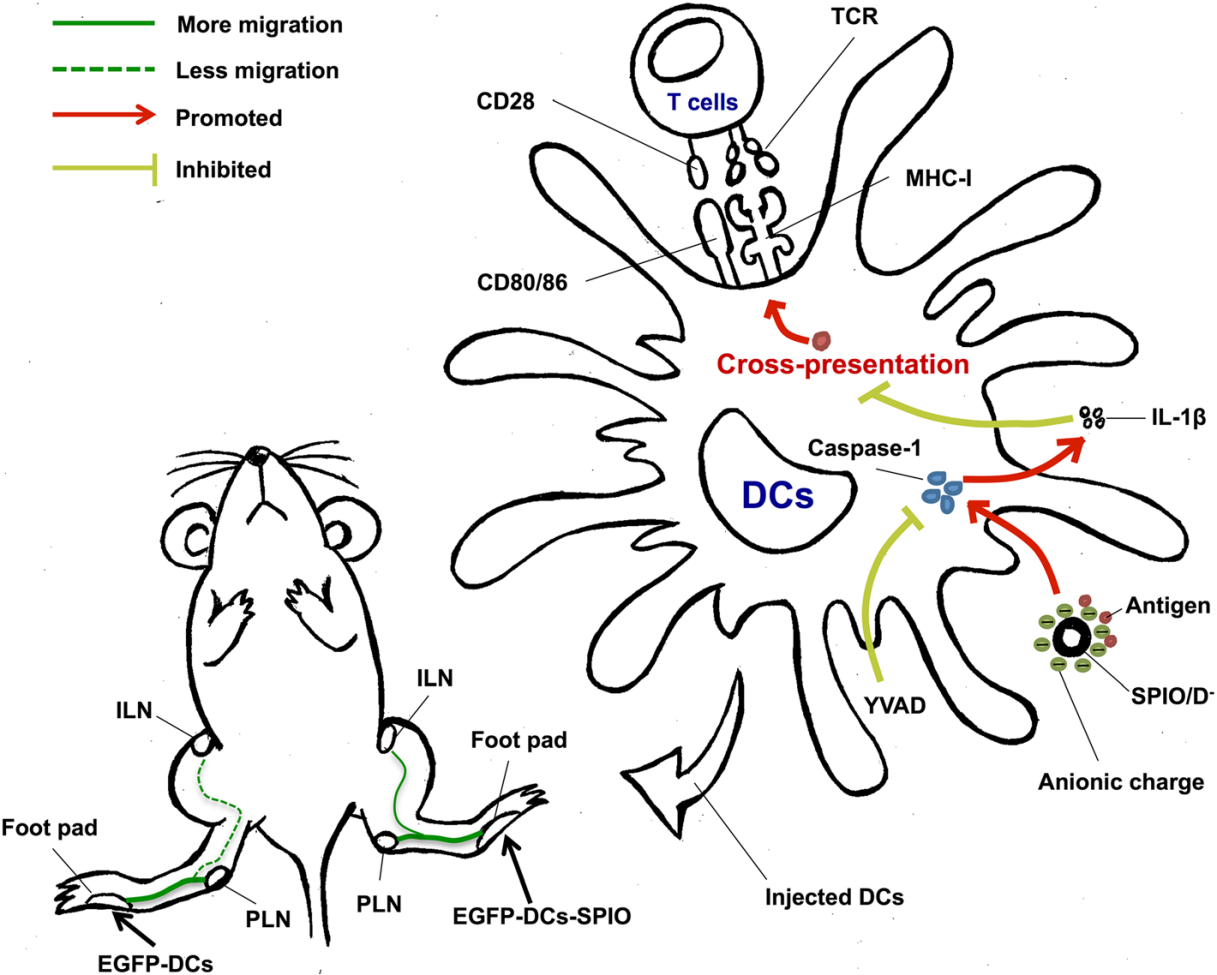
Graphical abstract of SPIO as a nano-adjuvant for DCs. SPIO enhances the function of DCs by promoting the loading of DCs; thus, SPIO-labeled DCs can migrate to the ILNs active in an immune organ and may offer a new approach in cancer immunotherapy. Anionic-charged SPIOs activate protective IL-1β responses by triggering caspase-1 in DCs, thereby impairing antigen presentation to active T cells

As shown in the graphical abstract in Fig. 6, SPIO can promote the maturation, migration, and cross-presentation of DCs. Moderate IL-1β activity is partly related to the antigen cross-presentation of DCs. In addition, negatively charged nanoparticles can activate excessive IL-1β and subsequently inhibit the functions of DCs. In summary, our results indicate that SPIO exhibit many biological properties and have promising adjuvant potential. These findings will help identify the optimal choice of nanoadjuvants for the development of DC vaccines in the future.

## Abbreviations

APCs: Antigen-presenting cells
APTS: 3-Aminopropyltrimethoxysilane
ATP: Adenosine triphosphate
CPRG: Chlorophenol red-β-d-galactopyranoside
CTL: Cytotoxic T cell
DAMPs: Damage-associated molecular patterns
DC: Dendritic cell
DMSA: Meso-2, 3-dimercaptosuccinic acid
EGFP: Enhanced green fluorescent protein
IL-1β: Interleukin-1β
MHC-I: Major histocompatibility complex class I
NLRP3: NACHT, LRR, and PYD domains-containing protein 3
OVA: Ovalbumin
PAMPs: Pathogen-associated molecular patterns
SPIO: Superparamagnetic iron oxide nanoparticles
SPIO/A^+^: SPIO coated with APTS
SPIO/D^−^: SPIO coated with DMSA
TLRs: Toll-like receptors
